# In vitro evaluation of *Curcuma longa* extract for antifungal, antibiofilm, and synergistic activity against *Candida* spp.

**DOI:** 10.1038/s41598-026-59895-9

**Published:** 2026-07-06

**Authors:** Hanaa F. Abdel-Aty, Abdullah M. Abdo, Reham M. Raafat Hamed

**Affiliations:** 1 Medical Microbiology and Immunology Department, Faculty of Medicine, Cairo University, Cairo, Egypt; 2 Botany and Microbiology Department, Faculty of Science, Al-Azhar University, Cairo, Egypt; 3 Medical Microbiology and Immunology Department, Faculty of Medicine, Badya University, Cairo, Egypt

**Keywords:** *Curcuma longa*, *Candida* spp., Antifungal activity, Biofilm inhibition, Fluconazole synergy, Amphotericin B, Minimal Inhibitory Concentration (MIC), Antifungal resistance, Drug discovery, Medical research, Microbiology

## Abstract

The rising incidence of non*-albicans Candida* (NAC) species and their developing resistance to traditional antifungal medications make candidiasis a significant problem in clinical microbiology. Another factor that leads to persistence and treatment failure is biofilm development. The purpose of this study was to assess the antifungal, antibiofilm, and synergistic properties of an ethanolic extract of *Curcuma longa (C. longa)*, which was dried and then reconstituted in distilled water, against clinical isolates of *Candida*, with chemical characterization confirmed by Fourier Transform Infrared (FT-IR) spectroscopy. Using the Vitek 2 technique, chromogenic agar, and the germ tube test (GTT), 40 *Candida isolates* were identified from a variety of clinical cases. Using the broth microdilution to detect Minimal Inhibitory Concentration (MIC) and disc diffusion techniques, antifungal susceptibility was assessed in compliance with CLSI (2022) recommendations. Combination disc testing was used to evaluate the synergy between *C. longa* extract and fluconazole or amphotericin B, and the crystal violet microtiter plate assay was used to examine the antibiofilm activity. The chemical composition of the extract was analyzed by FT-IR (Bruker TENSOR 37, Kalkar, Germany) in the 450–3500 cm⁻¹ range. *Candida* species that are non-*albicans* accounted for the majority (67.5%), with *C. glabrata* being the most common isolate (50%). The ethanolic extract of *C. longa* showed modest antifungal activity, with MIC values ranging from 6.4 to 815 µg/mL and mean inhibition zones of 12.33 ± 7.17 mm. The extract exhibited significant synergistic effects with fluconazole (*p* < 0.001) and amphotericin B (*p* = 0.05), resulting in enhanced inhibition zones when compared to the medicines alone. Significant antibiofilm properties were also shown by the extract, which, on average, prevented biofilm formation by 31.7%. FT-IR analysis revealed prominent absorption bands at 3424.55 cm^−1^ (O–H stretching, phenols), 1650.44 cm^−1^ (C = O stretching, amides), and 1161.08 cm^−1^ (C–O stretching, ethers), confirming the presence of bioactive functional groups such as polyphenols, curcuminoids, and terpenoids, which are associated with antifungal and antioxidant activity. *Curcuma longa*’s ethanolic extract, when reconstituted in distilled water, showed encouraging antifungal and antibiofilm properties and enhanced the effectiveness of common antifungal medications. These results point to *C. longa* extract as a viable natural adjuvant treatment option for treating *Candida* infections linked to biofilms and overcoming antifungal resistance. FT-IR characterization confirmed the presence of key functional groups related to curcumin derivatives, supporting its potential as a natural adjunctive therapeutic candidate for managing biofilm-associated *Candida* infections and combating antifungal resistance.

## Introduction

A major problem in both community and hospital settings, candidiasis is still one of the most common opportunistic fungal diseases in the world. The growing number of immunocompromised people worldwide, because of organ transplants, diabetes, cancer, and prolonged use of antibiotics or steroids, has led to a rise in the prevalence of invasive *Candida* infections^1,2^. Historically, *Candida albicans (C. albicans)* was the dominant etiological agent; however, in recent years, non-*albicans Candida* (NAC) species such as *Candida glabrata*  (*C. glabrata)*, *Candida tropicalis (C. tropicalis)*, *Candida krusei* (*C. krusei)*, and *Candida parapsilosis* (*C. parapsilosis)* have emerged as major pathogens, accounting for over 60% of clinical isolates in several regions^3,4^. These species are particularly concerning because many exhibit intrinsic or acquired resistance to azole and polyene antifungals^5^.

Several virulence factors, such as adhesion, phenotypic switching, and biofilm formation, increase the pathogenicity of *Candida* species^6–8^. Structured microbial communities known as biofilms are encased in an extracellular polymeric matrix, which increases their resilience to antifungal drugs and host defences^9^. Because of decreased drug penetration, changed gene expression, and efflux pump activation, fungal cells in biofilms can be up to a thousand times more resistant to azoles and amphotericin B than planktonic forms^10,11^. As a result, *Candida* biofilms are among the most resistant nosocomial infection types, particularly on medical equipment such as catheters and prosthetic implants^12,13^.

Even though a number of antifungal classes, including azoles, polyenes, echinocandins, and allylamines, have been developed, there are still few treatment alternatives accessible^14^. Their efficacy is restricted by host toxicity, pharmacokinetic unpredictability, and cross-resistance mechanisms. For example, amphotericin B retains its effectiveness as a fungicidal drug due to ergosterol binding and pore formation; nevertheless, its clinical use is restricted due to nephrotoxicity^15,16^. Liposomal formulations are still expensive and inaccessible in low-resource settings, even if their toxicity has decreased. Furthermore, an increasing number of NAC species, such as *Candida auris*, which possesses 30–60% amphotericin B resistance, have been reported to be resistant to fluconazole and amphotericin B^17,18^. Concerns that we are entering a “post-antibiotic” and “post-antifungal” era are supported by this pattern^19^.

In this regard, chemicals produced from plants have gained attention as potential adjuvants or substitutes in antifungal treatment. Polyphenols, terpenoids, and curcuminoids are examples of phytochemicals that have broad-spectrum antibacterial activity and frequently show less cytotoxicity to human cells^9,20^. When used with traditional antifungals, natural compounds can increase membrane permeability, block efflux pumps, and compromise the integrity of biofilms^6^.

Traditional medicine has traditionally utilized the Zingiberaceae family plant *Curcuma longa* (*C. longa* (to treat gastrointestinal issues, inflammation, and wound healing. Antimicrobial, anti-inflammatory, and antioxidant qualities are exhibited by curcumin, its primary bioactive constituent^21,22^. Its ability to inhibit *Candida* growth, hyphal formation, and biofilm development has been validated by numerous studies^23,24^. According to recent research, curcumin may have synergistic effects with fluconazole and amphotericin B, increasing their effectiveness against resistant isolates^25^.

In light of the growing incidence of resistant *Candida* infections and the shortcomings of existing antifungal treatments, it is pertinent and clinically relevant to assess natural extracts like *C. longa* as supplemental medicines. Determining the antifungal, antibiofilm, and synergistic properties of ethanolic *C. longa* extract reconstituted in distilled water against clinical isolates of *Candida* spp. was the goal of this investigation. The study aims to clarify the possible contribution of chemicals originating from plants to the fight against antifungal resistance linked to biofilms.

## Materials and methods

### Study design and ethical approval

This cross-sectional in vitro study was carried out at Cairo University’s Faculty of Medicine’s Department of Medical Microbiology and Immunology from January to July 2025. From a variety of specimens, including urine, blood, pus, sputum, and endotracheal aspirates obtained from the diagnostic laboratories of Kasr Al-Ainy University Hospital, forty clinical *Candida* isolates were obtained. The research was authorized by Cairo University’s Faculty of Medicine’s Research Ethics Committee (Approval No. N-376-2024) and conducted in compliance with the 2013 Declaration of Helsinki. The requirement for informed consent was waived by the Research Ethics Committee because this was retrospective research involving anonymized microbial isolates.

### Plant material source and authorization

The plant material used in this research, *C. longa L.*, was obtained under official authorization from the Botany and Microbiology Department, Faculty of Science, Al-Azhar University, Cairo, Egypt, under permission code AZU/SCI/BOT/2025-057.

The collection and use of *C. longa* complied with national and institutional guidelines on biodiversity conservation and scientific research. The specimen is publicly accessible for future reference in accordance with herbarium policies.

### Identification of *Candida* isolates

Specimens were cultured on Sabouraud Dextrose Agar (SDA) (Oxoid, UK) and incubated at 37 °C for 24–72 h. Yeast morphology was confirmed microscopically after Gram staining. Presumptive identification was performed by the Germ Tube Test (GTT) for differentiation of *C. albicans* and *C. dubliniensis*^26^.

Isolates were further identified using HiCrome™ *Candida* Differential Agar (HiMedia, India), incubated at 30 °C for 48 h, and color-coded according to the manufacturer’s instructions. Final confirmation was obtained using the Vitek 2 Compact System (bioMérieux, France).

### Preparation of ethanolic *Curcuma longa* extract

The ethanolic extract of *C. longa L.* rhizomes was prepared according to Subramaniam et al. (2022)^27^ with slight modifications. Briefly, 5.00 g of finely powdered *C. longa* rhizomes was mixed with 20 mL of absolute ethanol (corrected from 2 mL; see note below) in a sterile, sealed container. The mixture was left to stand at room temperature (25 ± 2 °C) for five days with intermittent shaking to ensure efficient extraction of bioactive compounds.

After incubation, the mixture was centrifuged at 3,870 rpm for 10 min, and the process was repeated twice to remove solid residues. The clear supernatant was collected, and the solvent was evaporated at 50 °C in a hot-air oven to yield the dry extract.

The extraction yield was calculated using the formula:

$$\:\mathrm{D}\mathrm{r}\mathrm{y}\:\mathrm{e}\mathrm{x}\mathrm{t}\mathrm{r}\mathrm{a}\mathrm{c}\mathrm{t}\:\mathrm{r}\mathrm{e}\mathrm{c}\mathrm{o}\mathrm{v}\mathrm{e}\mathrm{r}\mathrm{e}\mathrm{d}\:=\:\mathrm{I}\mathrm{n}\mathrm{i}\mathrm{t}\mathrm{i}\mathrm{a}\mathrm{l}\:\mathrm{m}\mathrm{a}\mathrm{s}\mathrm{s}\:-\:\mathrm{D}\mathrm{e}\mathrm{b}\mathrm{r}\mathrm{i}\mathrm{s}\:$$

$$\:=5.00\:\mathrm{g}\:-\:3.37\:\mathrm{g}\:=\:1.63\:\mathrm{g}$$

**Fig. 1:**
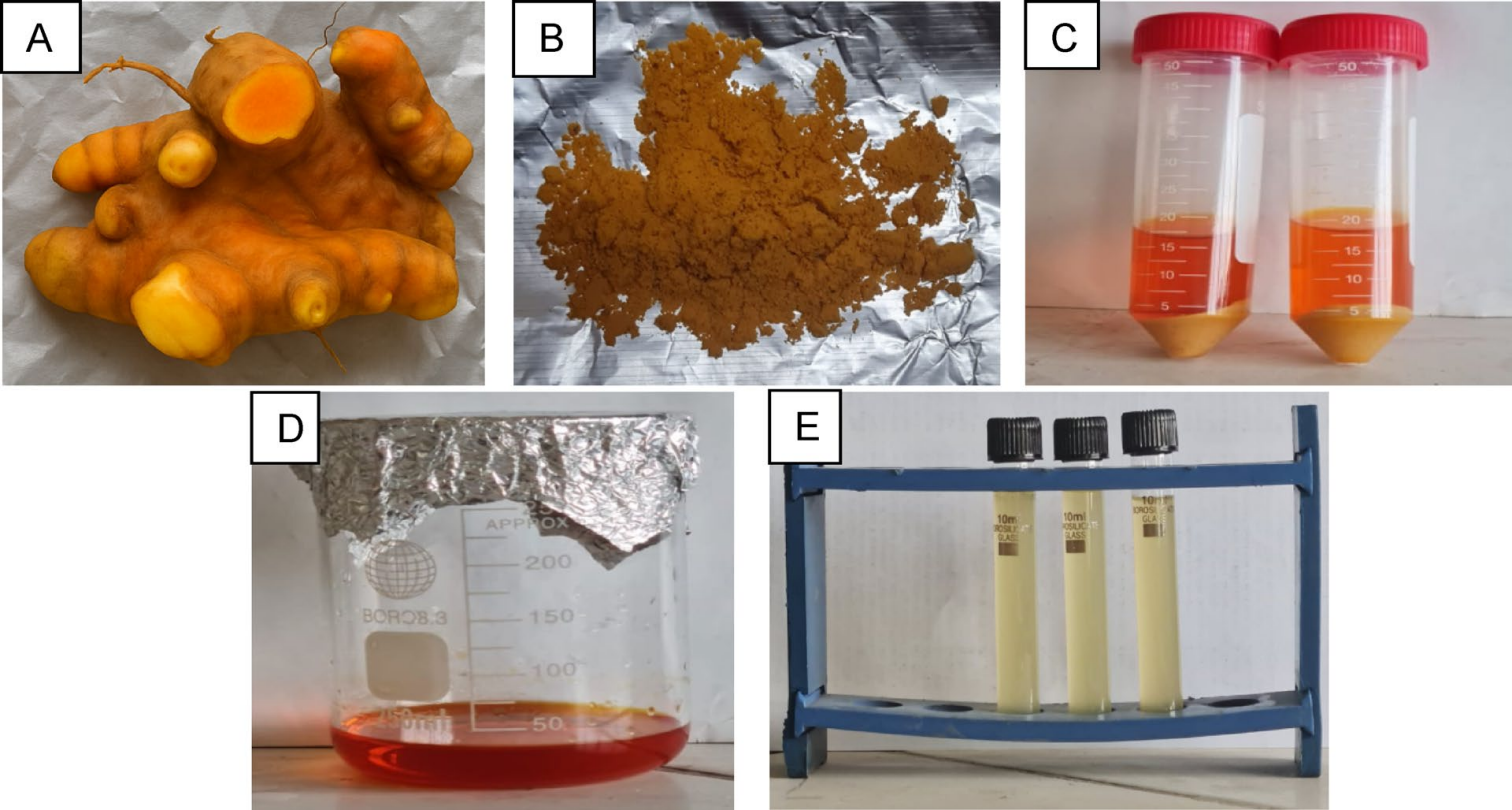
Preparation steps of the ethanolic extract of *C. longa*. (**A**) Fresh rhizomes of *C. longa* collected under authorized botanical permission. (**B**) Dried and powdered *C. longa* rhizome used for extraction. (**C**) Initial maceration of *C. longa* powder in absolute ethanol. (**D**) Clear ethanolic supernatant obtained after centrifugation and filtration before solvent evaporation. (**E**) Final dried ethanolic extract residue reconstituted in sterile distilled water to prepare the stock solution (1.63 mg/mL) used for antifungal and antibiofilm testing.

The dried extract was reconstituted in sterile distilled water to prepare a stock solution (1.63 mg/mL), sterilized by filtration through a 0.22 μm cellulose acetate filter (Millipore, Billerica, MA, USA), and stored at 4 °C until use in biological assays (Fig. 1).

### FT-IR spectrum analysis of ethanolic extract of *Curcuma longa*

For Fourier-transform infrared (FT-IR) spectroscopy, a separate ethanolic extract was prepared via Soxhlet extraction to ensure maximum compound recovery and purity for spectral analysis. Exactly 5.00 g of powdered *C. longa* rhizomes was subjected to extraction using 95% ethanol in a Soxhlet apparatus for 8 h.

The obtained extract was concentrated under reduced pressure using a rotary evaporator and stored in an airtight container until analysis.

FT-IR spectra were recorded using a Bruker TENSOR 37 FT-IR spectrophotometer (Kalkar, Germany) with KBr pellets as the medium. The spectra were acquired in the wavenumber range 450–3500 cm⁻¹ with a resolution of 4 cm⁻¹. The characteristic absorption bands were interpreted to identify the functional groups present in the extract^28,29^.

### Antifungal susceptibility testing

For antifungal susceptibility testing, Mueller–Hinton agar supplemented with 2% glucose was used in accordance with CLSI (2022; M44-S3) guidelines^30^ to ensure standard comparability of inhibition zones. to improve yeast growth and produce sharper inhibition zones, as detailed in standards like M44. This enriched medium supports consistent growth and allows for standardized reading of zones after 24–48 h at 35 °C. Each isolate was adjusted to 0.5 McFarland (≈ 1 × 10⁶ CFU/mL) and spread onto Mueller–Hinton Agar supplemented with 2% glucose. Discs containing fluconazole (25 µg), amphotericin B (20 U), and *C. longa* extract (10 µL/disc) were placed onto the inoculated plates and incubated at 35 °C for 24–48 h.

For synergism testing, 10 µL of *C. longa* extract was added to fluconazole or amphotericin B discs before placement. Synergism was defined as a statistically significant increase in inhibition zone diameter of the antifungal agent in combination with *C. longa* extract compared with the antifungal agent alone, supported by paired statistical analysis. This operational definition is consistent with disc diffusion–based combination screening methods in *Candida* studies, where synergism is inferred from a significant increase in inhibition zones in combination relative to individual agents (Maxwell et al., 2021)^31^. Inhibition zones were measured in millimeters (mm).

### Determination of minimum inhibitory concentration (MIC)

MIC values were determined by the broth microdilution method in sterile 96-well microplates, as described by Karpiński et al. (2023)^32^. Twofold serial dilutions of *C. longa* extract (1630–0.8 µg/mL) were prepared in Tryptic Soy Broth (TSB) with 0.5% glucose, which was used to ensure methodological consistency across antifungal, biofilm, and plant‑extract assays. TSB has been reported in the literature as a suitable alternative to RPMI 1640 recommended by CLSI, and has been successfully employed in published MIC studies for *Candida spp*. Enriched media have been widely used in comparative phytochemical antifungal screening, particularly when the objective is to compare relative inhibitory activity rather than assign clinical breakpoints, and 100 µL of standardized *Candida* suspension (0.5 McFarland) was added to each well. After incubation at 37 °C for 48 h, the MIC was recorded as the lowest concentration showing no visible growth. Activity was classified as: ≤1 µg/mL (highly active), 1–10 µg/mL (significant), 10–100 µg/mL (moderate), and 100–1000 µg/mL (low)^33^. The microdilution process was carried out in accordance with CLSI M27 guidelines^34^ with regard to inoculum preparation, incubation conditions, and endpoint determination in order to guarantee methodological uniformity and comparability with antifungal susceptibility testing for yeasts. However, there are no CLSI clinical breakpoints for this extract. Therefore, rather than clinical susceptibility, the extract MIC values were interpreted as relative inhibitory activity.

## Biofilm formation assay

Biofilm formation was quantified by the microtiter plate method^10^ Standardized *Candida* suspensions (0.5 McFarland, diluted 1:20, ≈ 1.5 × 10⁶ CFU/mL) were inoculated into 96-well flat-bottom microplates (180 µL broth + 20 µL inoculum) and incubated at 37 °C for 24 h. Wells were gently washed with PBS, air-dried, fixed with methanol for 15 min, and stained with 1% crystal violet for 15 min. The bound dye was dissolved in 95% ethanol, and to measure total biofilm biomass, a commonly used comparison endpoint in antifungal antibiofilm studies, crystal violet was chosen. Despite the potential for metabolic viability dyes to offer supplementary functional information, biomass quantification is still a reliable and repeatable primary sign of biofilm inhibition that enables consistent comparison across experimental settings. All assays were performed in independent triplicate experiments^35^.

Then the absorbance was measured at 570 nm. The cut-off optical density (OD₍c₎) was defined as:

$$\:\mathrm{O}\mathrm{D}\left(\mathrm{c}\right)=\mathrm{O}\mathrm{D}\:\mathrm{o}\mathrm{f}\:\mathrm{n}\mathrm{e}\mathrm{g}\mathrm{a}\mathrm{t}\mathrm{i}\mathrm{v}\mathrm{e}\:\mathrm{c}\mathrm{o}\mathrm{n}\mathrm{t}\mathrm{r}\mathrm{o}\mathrm{l}\text{}+(2\times\:\mathrm{S}\mathrm{D}\:\mathrm{o}\mathrm{f}\:\mathrm{n}\mathrm{e}\mathrm{g}\mathrm{a}\mathrm{t}\mathrm{i}\mathrm{v}\mathrm{e}\:\mathrm{c}\mathrm{o}\mathrm{n}\mathrm{t}\mathrm{r}\mathrm{o}\mathrm{l})\:$$

Isolates with OD values greater than OD₍c₎ were considered biofilm producers^26^.

### Biofilm inhibition assay

Only biofilm-positive isolates were selected for antibiofilm assays to ensure measurable baseline biofilm mass and valid inhibition quantification. A standard microtiter plate method was used to evaluate biofilm development and inhibition. Since *Candida* suspensions were co-incubated with the extract at the beginning of biofilm growth, surface attachment was possible prior to extracellular matrix maturation, which naturally included an adhesion phase in the experimental design. Rather than disrupting preexisting biofilms, this design is commonly used in vitro models to assess interference with early biofilm development.

The inhibitory effect of *C. longa* extract on biofilm formation (not preformed biofilms) by exposing *Candida* inoculum to the extract during the initial adhesion and growth phase was tested according to El-Bashiti et al. (2019)^36^. Each well received 180 µL of *C. longa* extract and 20 µL of standardized inoculum, followed by incubation at 37 °C for 48 h. After washing and staining, absorbance was measured at 570 nm. The percentage inhibition of biofilm was calculated as:

$${\text{Biofilm Inhibition }}\left( {{\% }} \right) = \left( {1 - {\text{}}\frac{{{\mathrm{OD}}_{{\mathrm{t}}} }}{{{\mathrm{ODc}}}}} \right) \times 100$$

Where OD_t_ = absorbance of treated wells and ODc = control wells without extract.

### Statistical analysis

For statistical analysis, SPSS v28.0 (IBM Corp., USA) was used. Data distribution was assessed prior to hypothesis testing in order to verify that non-parametric approach was appropriate. In order to guarantee the robustness of antibiofilm interpretation, replicate variability was investigated and significance criteria were confirmed. By doing these actions, analytical bias is reduced and statistical dependability is strengthened. Frequency and percentage were used to represent qualitative variables, whereas mean ± SD or median (range) were used to represent quantitative data. Because of the non-normal distribution of OD values, non-parametric tests were used to analyze the biofilm inhibition data. When applicable, the Mann-Whitney U and Kruskal-Wallis tests were used. P-values less than 0.05 were regarded as statistically significant^34^.

Fisher’s exact test or the Chi-square (χ²) test evaluated categorical relationships^35^. Using Spearman’s correlation coefficient, correlations between inhibition zones, MICs, and biofilm inhibition were examined^36^. Diagnostic performance indices were computed following Galen (1980) using the following Equations.

$$\:\mathrm{S}\mathrm{e}\mathrm{n}\mathrm{s}\mathrm{i}\mathrm{t}\mathrm{i}\mathrm{v}\mathrm{i}\mathrm{t}\mathrm{y}=\frac{\left(\:\mathrm{N}\mathrm{u}\mathrm{m}\mathrm{b}\mathrm{e}\mathrm{r}\:\mathrm{o}\mathrm{f}\:\mathrm{t}\mathrm{r}\mathrm{u}\mathrm{e}\:\mathrm{p}\mathrm{o}\mathrm{s}\mathrm{i}\mathrm{t}\mathrm{i}\mathrm{v}\mathrm{e}\:\mathrm{r}\mathrm{e}\mathrm{s}\mathrm{u}\mathrm{l}\mathrm{t}\mathrm{s}\:\right(\mathrm{T}\mathrm{P}\left)\:\:\mathrm{X}100\right)}{(\mathrm{T}\mathrm{P}+\mathrm{F}\mathrm{N})}$$

$$\:\mathrm{S}\mathrm{p}\mathrm{e}\mathrm{c}\mathrm{i}\mathrm{f}\mathrm{i}\mathrm{c}\mathrm{i}\mathrm{t}\mathrm{y}=\frac{\left(\:\mathrm{N}\mathrm{u}\mathrm{m}\mathrm{b}\mathrm{e}\mathrm{r}\:\mathrm{o}\mathrm{f}\:\mathrm{t}\mathrm{r}\mathrm{u}\mathrm{e}\:\mathrm{n}\mathrm{e}\mathrm{g}\mathrm{a}\mathrm{t}\mathrm{i}\mathrm{v}\mathrm{e}\:\mathrm{r}\mathrm{e}\mathrm{s}\mathrm{u}\mathrm{l}\mathrm{t}\mathrm{s}\:\right(\mathrm{T}\mathrm{N}\left)\:\mathrm{X}100\right)}{(\mathrm{T}\mathrm{N}+\mathrm{F}\mathrm{P})}$$

$$\:\mathrm{P}\mathrm{o}\mathrm{s}\mathrm{i}\mathrm{t}\mathrm{i}\mathrm{v}\mathrm{e}\:\mathrm{p}\mathrm{r}\mathrm{e}\mathrm{d}\mathrm{i}\mathrm{c}\mathrm{t}\mathrm{i}\mathrm{v}\mathrm{e}\:\mathrm{v}\mathrm{a}\mathrm{l}\mathrm{u}\mathrm{e}=\frac{\left(\:\mathrm{N}\mathrm{u}\mathrm{m}\mathrm{b}\mathrm{e}\mathrm{r}\:\mathrm{o}\mathrm{f}\:\mathrm{t}\mathrm{r}\mathrm{u}\mathrm{e}\:\mathrm{p}\mathrm{o}\mathrm{s}\mathrm{i}\mathrm{t}\mathrm{i}\mathrm{v}\mathrm{e}\:\mathrm{r}\mathrm{e}\mathrm{s}\mathrm{u}\mathrm{l}\mathrm{t}\mathrm{s}\:\right(\mathrm{T}\mathrm{P}\left)\:\:\mathrm{X}100\right)}{(\mathrm{T}\mathrm{P}+\mathrm{F}\mathrm{P})}$$

$$\:\mathrm{N}\mathrm{e}\mathrm{g}\mathrm{a}\mathrm{t}\mathrm{i}\mathrm{v}\mathrm{e}\:\mathrm{p}\mathrm{r}\mathrm{e}\mathrm{d}\mathrm{i}\mathrm{c}\mathrm{t}\mathrm{i}\mathrm{v}\mathrm{e}\:\mathrm{v}\mathrm{a}\mathrm{l}\mathrm{u}\mathrm{e}\:=\frac{\left(\:\mathrm{N}\mathrm{u}\mathrm{m}\mathrm{b}\mathrm{e}\mathrm{r}\:\mathrm{o}\mathrm{f}\:\mathrm{t}\mathrm{r}\mathrm{u}\mathrm{e}\:\mathrm{n}\mathrm{e}\mathrm{g}\mathrm{a}\mathrm{t}\mathrm{i}\mathrm{v}\mathrm{e}\:\mathrm{r}\mathrm{e}\mathrm{s}\mathrm{u}\mathrm{l}\mathrm{t}\mathrm{s}\:\right(\mathrm{T}\mathrm{N}\left)\:\:\mathrm{X}100\right)}{(\mathrm{T}\mathrm{N}+\mathrm{F}\mathrm{N})}$$

$$\:\mathrm{O}\mathrm{v}\mathrm{e}\mathrm{r}\mathrm{a}\mathrm{l}\mathrm{l}\:\mathrm{a}\mathrm{c}\mathrm{c}\mathrm{u}\mathrm{r}\mathrm{a}\mathrm{c}\mathrm{y}=\frac{\left(\:\right(\mathrm{T}\mathrm{P}\:+\mathrm{T}\mathrm{N}\left)\:\:\mathrm{X}100\right)}{(\mathrm{T}\mathrm{P}\:+\:\mathrm{T}\mathrm{N}\:+\:\mathrm{F}\mathrm{P}\:+\:\mathrm{F}\mathrm{N}\:)}$$

A *P-value* < 0.05 was considered statistically significant for all analyses.

## Results

The present study included 40 *Candida* isolates (13 (32.5%) isolates were *C. albican*, and 27 (67.5%) isolates were NAC, 20 (74%) *C. glabrata*, 3 (11%) *C. tropicalis*, 3 (11%) *C. krusei*, and one (3%) *C. parapsilosis*). Out of the 40 *Candida* isolates, 23 (57.5%) were from female patients and 17 (42.5%) from male patients. The majority of the isolates (35%) were from the urine, followed by blood and pus (15%), sputum (12.5%), and endotracheal tube aspirate (10%).

According to GTT, 18 (45%) isolates were germ tube positive, and 22(55%) isolates were germ tube negative. Confirmation of species identification was done using the Vitek 2 compact system and revealed that *C. glabrata* was the most frequently isolated species (20/40, 50%). All *C. albicans* isolates (13 isolates, 100%) identified by the HiCrome differential agar medium and Vitek 2 system were positive by GTT. Whereas 22 (81.5%) of the 27 NAC isolates identified by Vitek 2 showed negative GTT results, while 5 isolates (18.5%) showed false positive results (three isolates *C. glabrata*, one isolate *C. tropicalis*, and one isolate *C. krusei)*. Thus, GTT showed an overall sensitivity of 100%, specificity of 81.48%, PPV of 72.22%, NPV of 100%, and accuracy of 87.5%.

Subculturing on HiCrome agar showed different colors with respect to the diverse species of *Candida*. All the isolates that didn’t show filamentous growth and yielded different colors other than *C. albicans* (green color) were considered NAC spp. For *C. glabrata*, 18(90%) isolates produced the expected white creamy color, while 2 (10%) isolates produced a pale purple color. All 3 *C. tropicalis* isolates (100%), 3 *C. krusei isolates* (100%), and one *C. parapsilosis* (100%) isolate showed the expected reference color (Blue for *C. tropicalis*, purple for *C. krusei*, and white for *C. parapsilosis*). Thus, HiCrome differential agar medium showed an overall accuracy of 92.6% in the case of NAC spp.

According to CLSI (2022)^30^, *C. albicans*, *C. tropicalis*, and *C. parapsilosis* breakpoints for fluconazole are (sensitive if ≥ 17 mm; resistant if ≤ 13 mm). *C. glabrata* and *C. krusei* are intrinsically resistant to fluconazole; therefore, testing of the synergistic activity against them was ruled out. For amphotericin B breakpoint, *C. albicans*, *C. tropicalis*, *C. parapsilosis*, and *C. krusei* are considered sensitive if ≥ 15 mm and resistant if ≤ 10 mm. There are no interpretative breakpoint criteria available from CLSI for amphotericin B for *C. glabrata*. Accordingly, the seventeen *Candida* isolates tested for fluconazole showed sensitivity in 16 (94.1%) isolates and resistance in one (5.9%). Whereas 4 (20%) of the twenty *Candida* isolates tested for amphotericin B showed resistance, 2/13 (15.38%) isolates of *C. albicans*, 1/3 (33.33%) isolate of *C. tropicalis*, and 1/3 (33.33%) isolate of *C. krusei.*

**Fig. 2:**
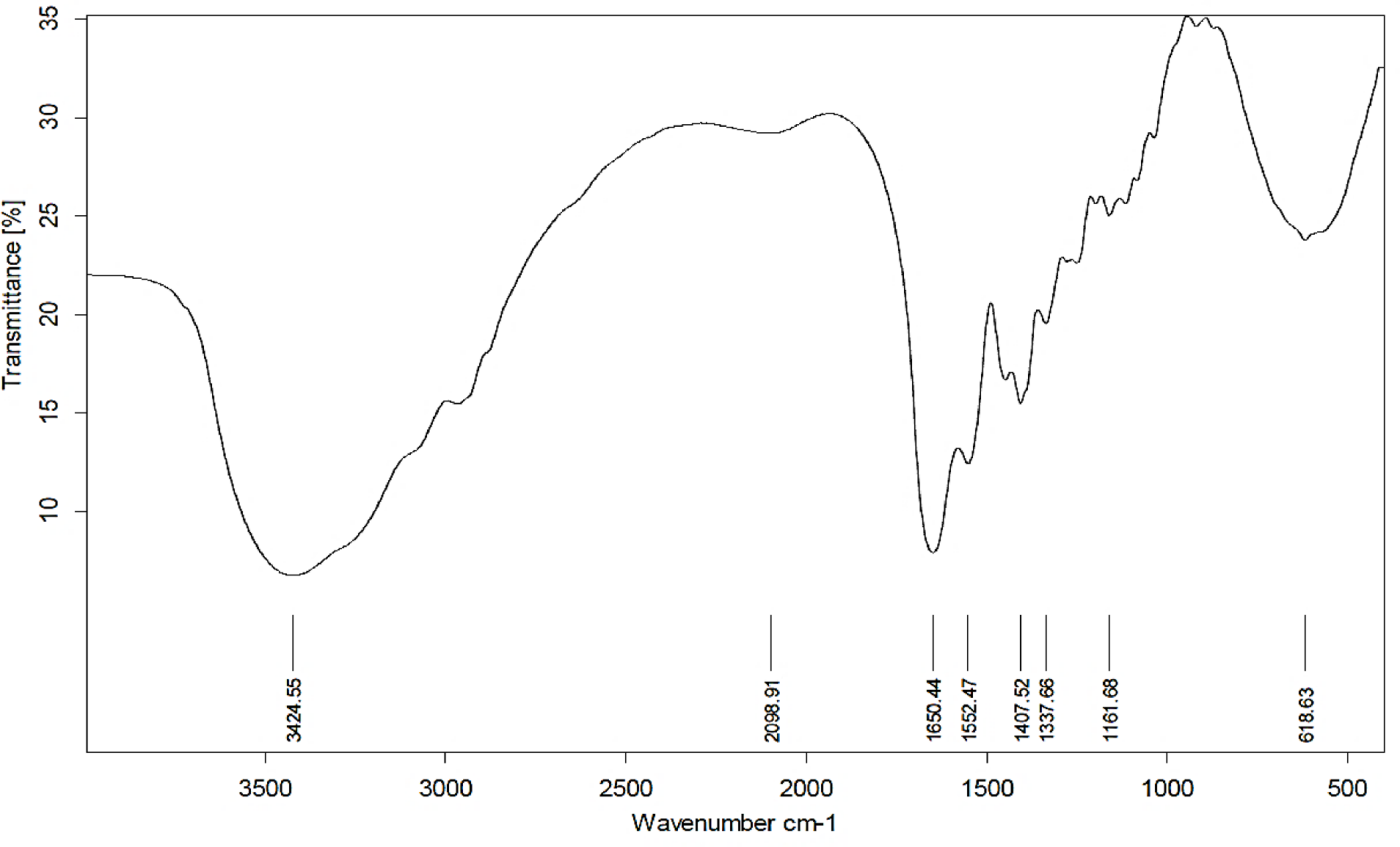
FT-IR spectrum of ethanolic extract of *C. longa.*

The FT-IR spectrum of *C. longa* extract (Fig. 2) exhibited several distinct absorption peaks indicating the presence of multiple functional groups associated with phytochemicals such as curcuminoids, phenolics, and terpenoids^37,38^ The broad band at 3424.55 cm⁻¹ corresponds to O–H stretching vibrations, indicating the presence of hydroxyl groups in alcohols and phenols. The weak band at 2098.91 cm⁻¹ may be attributed to C ≡ C stretching of alkynes. The strong peaks at 1650.44 cm⁻¹ and 1552.47 cm⁻¹ correspond to C = O stretching and N–H bending, respectively, confirming the presence of amide and carbonyl groups. The absorption bands observed at 1407.52 cm⁻¹ and 1337.66 cm⁻¹ suggest C–H bending and O–H deformation, while 1161.08 cm⁻¹ and 618.63 cm⁻¹ are indicative of C–O stretching and C–H bending vibrations typical of aromatic compounds. These findings confirmed the presence of functional groups commonly associated with compounds that contribute to the antimicrobial activity of *C. longa* extract.

As revealed in Table 1, the mean of the inhibitory zone diameter of *C. longa* extract against all tested *Candida* isolates was 12.33 ± 7.17 mm. It was observed that the *C. longa* zone diameter for 17 *Candida* isolates that tested with fluconazole (mean of *C. longa* inhibitory zones = 13.59 ± 9.45 mm) was significantly smaller than the inhibitory zone diameter of fluconazole (mean of fluconazole inhibitory zones = 28.06 ± 9.01, *P-value* = 0.0001). However, this diameter was significantly increased by the synergistic effects of *C. longa* extract when added to fluconazole, with a statistically highly significant difference (mean zone diameter = 31.94 ± 6.99, *P-value* < 0.0001). On the other hand, there was no statistically significant difference in the mean zone diameter of fluconazole when combined with *C. longa* extract (*P-value* = 0.1703).

**Fig. 3:**
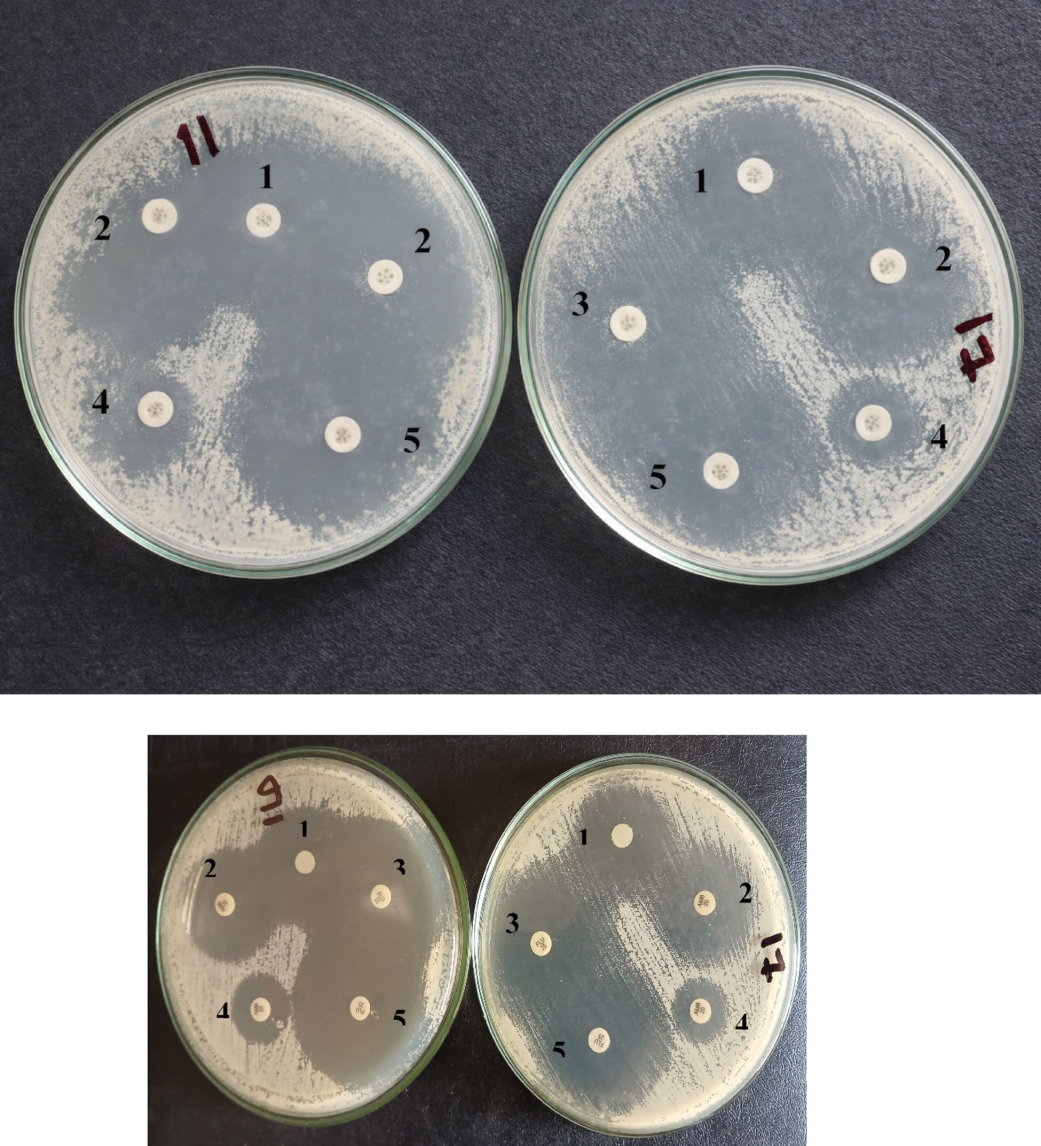
Antifungal susceptibility and synergism between *C. longa* extract and each of fluconazole and amphotericin B against *C. albicans*. 1: Zone inhibition of *C. longa* extract, 2: Zone inhibition of amphotericin B combined with *C. longa* extract, 3: Zone inhibition of fluconazole combined with *C. longa* extract, 4: Zone inhibition of amphotericin B, 5: Zone inhibition of fluconazole.

**Fig. 4:**
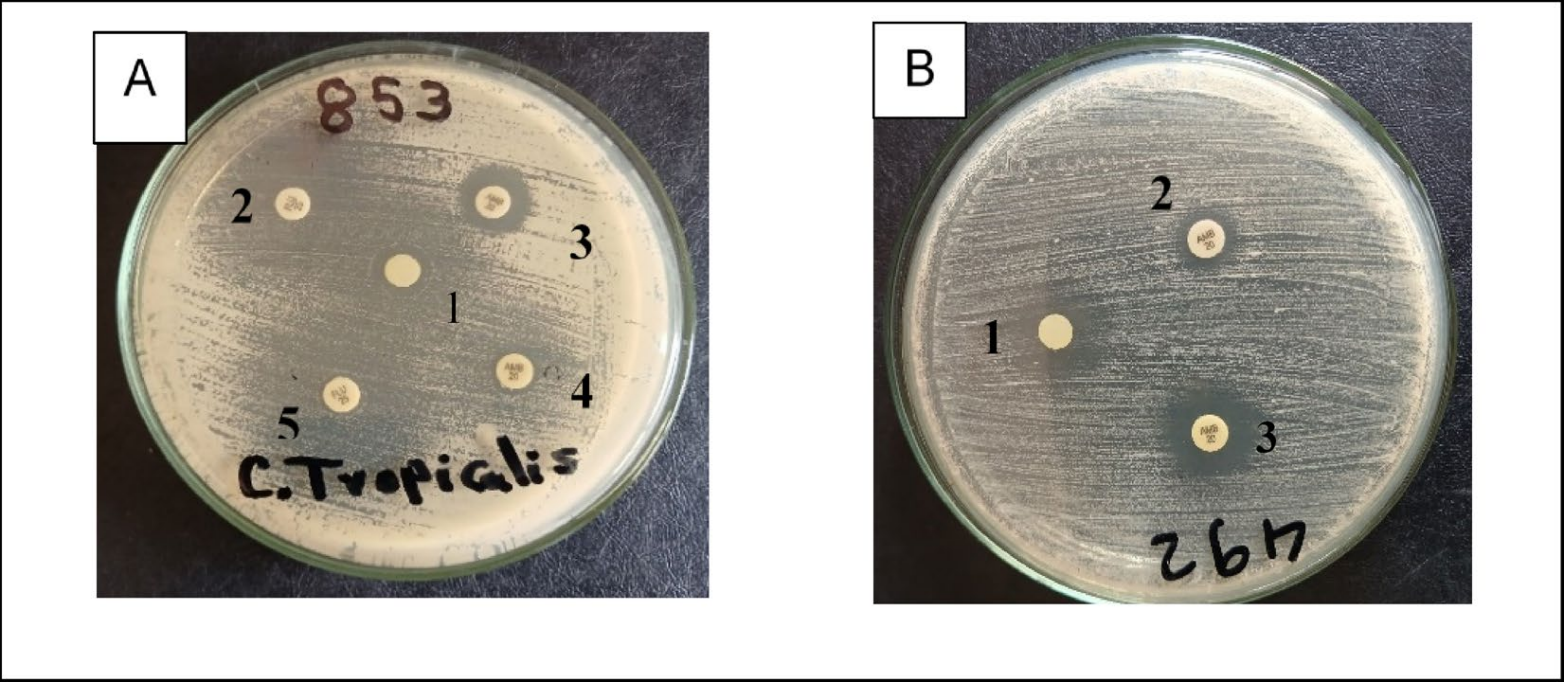
Antifungal susceptibility and synergism between *C. longa* extract and each of fluconazole and amphotericin B against NAC spp. (**A**) *C. tropicalis*, 1: Zone inhibition of *C. longa* extract, 2: Zone inhibition of amphotericin B, 3: Zone inhibition of amphotericin B combined with *C. longa* extract, 4: Zone inhibition of fluconazole combined with *C. longa* extract, 5: Zone inhibition of fluconazole. (**B**) *C. glabrata*, 1: Zone inhibition of *C. longa* extract, 2: Zone inhibition of amphotericin B, 3: Zone inhibition of amphotericin B combined with *C. longa* extract.

On the other hand, the inhibitory zone diameter of *C. longa* extract in the 20 *Candida* isolates (mean of *C. longa* inhibitory zones = 12.45 ± 9.10) was smaller than the diameter of the inhibitory zone of amphotericin B, but with no statistically significant difference (mean of amphotericin B inhibitory zones = 14.20 ± 6.76, *P-value* = 0.4942. However, amphotericin B inhibitory zone diameter was increased by the synergistic effects of *C. longa* extract when added to it with no statistically significant difference (mean zone diameter = 17.75 ± 7.89, *P-value* = 0.1348. On the other hand, the inhibitory zone diameter of *C. longa* extract significantly increased when combined with amphotericin B, *P-value* = 0.05 (Figs. 3 and 4).

**Table 1:** The inhibition zone diameters exhibited by the tested agents against the tested *Candida* spp. (*N* = 40 isolates).

Mean ± SD of the inhibitory zones in millimeters				*P*-value
Fluconazole alone	28.06 ± 9.01 mm	*C. longa* extract	13.59 ± 9.45 mm	0.001**
		Fluconazole + *C. longa* extract	31.94 ± 6.99 mm	0.1703
Amphotericin B alone	14.20 ± 6.76 mm	*C. longa* extract	12.45 ± 9.10 mm	0.4942
		Amphotericin B + *C. longa* extract	17.75 ± 7.89 mm	0.1348
*C. longa* extract alone (40 isolates)	12.33 ± 7.17 mm	Fluconazole + *C. longa* extract	31.94 ± 6.99 mm	< 0.0001**
*C. longa* extract alone (17 isolates)	13.59 ± 9.45 mm	Amphotericin B + *C. longa* extract	17.75 ± 7.89 mm	0.05*
*C. longa* extract alone (20 isolates)	12.45 ± 9.10 mm			
Fluconazole + *C. longa* extract	31.94 ± 6.99	Amphotericin B + *C. longa* extract	17.75 ± 7.89 mm	0.001**

* Statistically significant at P < 0.05.

** Statistically highly significant at P < 0.001.

In the present study, it was observed that *C. longa* extract exhibited non-significantly higher antifungal activity against *C. albicans* than NAC spp. (*P-value* = 0.408) (Table 2).

**Table 2:** The antifungal activity of *C. longa* extract against the tested *C. albicans* and NAC spp.

	*Candida* spp				
	*C. albicans*		Non-*albicans Candida*		*P-value*
	Mean	SD	Mean	%	
*C. longa* extract (mm)	13.69	10.49	11.66	4.98	0.408
*C. longa* MIC (µg/mL)	183.74	314.91	280.51	297.85	0.147

Statistically significant at P< 0.05.

*C. longa* extract showed MIC values ranging from 6.40 to 815 µg/mL, with a mean MIC value = 252.29 ± 299.34 µg/mL. It presented a significant activity with a MIC value of 6.40 µg/mL against two isolates of *C. albicans* and one isolate of NAC spp. Additionally, it presented moderate activity with MICs ranging from 12.7 to 50.8 µg/mL, against 3 isolates of *C. albicans* and 6 isolates of NAC spp. Moreover, it showed low activity with MICs ranging from 101.8 to 815 µg/mL, against 2 isolates of *C. albicans* and 10 isolates of NAC spp. As revealed in Table 2, *C. longa* extract exhibited non-statistically significantly higher antifungal activity against *C. albicans*, mean MIC value = 183.74 ± 314.91 µg/mL, than NAC spp., mean MIC value = 280.51 ± 297.85 µg/mL (*P-value* = 0.147).

It was observed that the MIC values were significantly related to the inhibitory zone diameter of *C. longa* extract (*P-value* = < 0.001), as shown in Table 3.

**Table 3:** Relation between *C. longa* extract MIC and inhibitory zone diameters.

	*C. longa* MIC(µg/mL)						*P-value*
	Significant activity		Moderate activity		Low activity		
	Mean	SD	Mean	SD	Mean	SD	
*C. longa* extract (mm)	27.00	6.93	19.22	4.32	13.00	2.00	< 0.001

Statistically significant at P < 0.05.

Statistically highly significant at P < 0.001.

### Biofilm formation assay

Among the 40 *Candida* isolates, 16 (40%) were biofilm producers, 5/13 (38.5%) *C. albicans*, 1/3 (33.3%) *C trobicalis*, 2/3 (66.7%) *C. kruesi*, 8/20 (40.0%) *C. glabrata*, Table 4. Biofilm producer isolates tested in this study were selected for the antibiofilm assay.

**Table 4:** Biofilm formation among different *Candida* spp.

	*C. albicans*		*C.tropicalis*		*C.parapslosis*		*C.kruesi*		*Cglaprata*	
	Count	%	Count	%	Count	%	Count	%	Count	%
Biofilm producer *Candida* spp.	5	38.5	1	33.3	0	0.0	2	66.7	8	40.0

### Biofilm inhibition assay

**Fig. 5:**
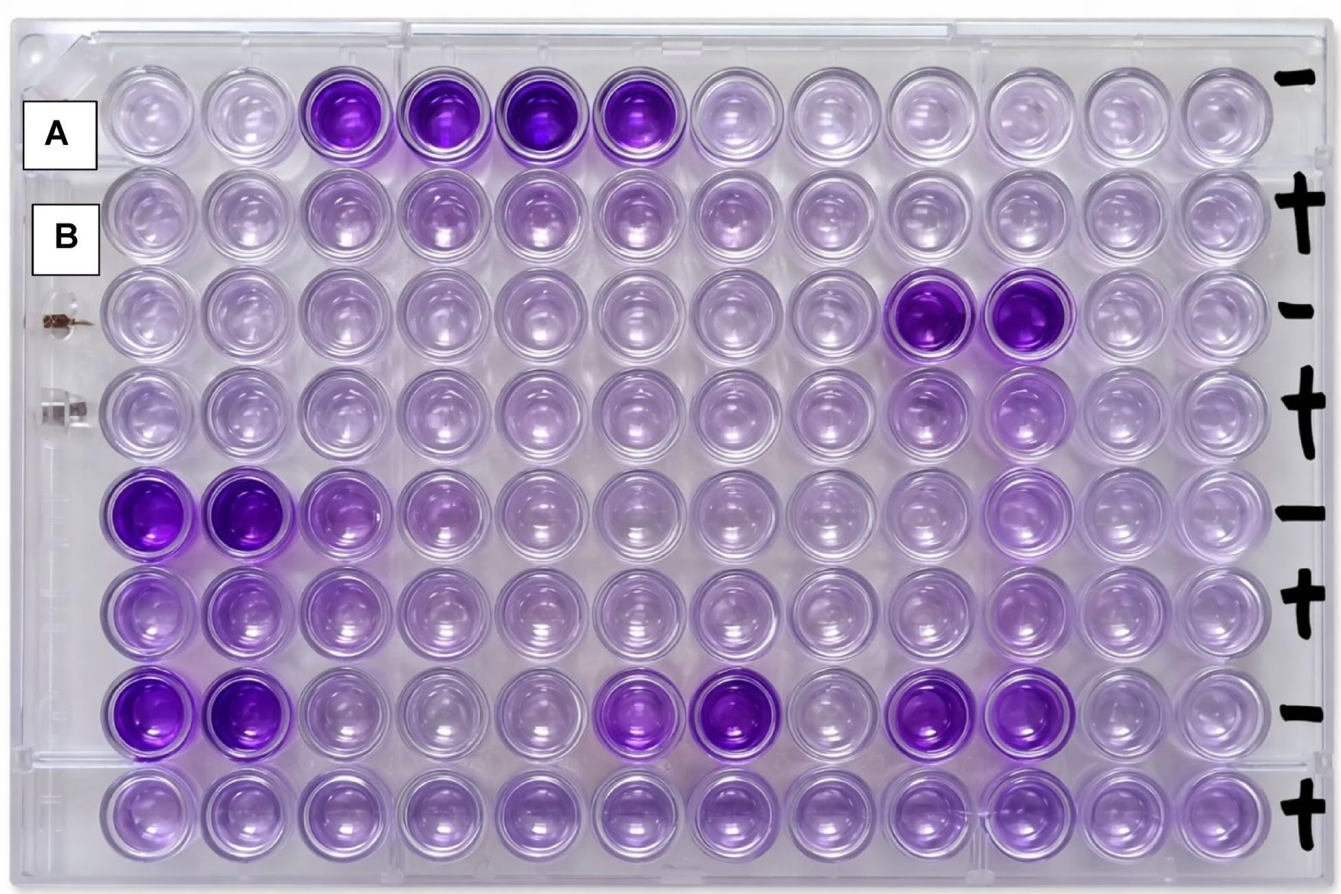
Biofilm inhibition activity of *C. longa* extract, (**A**) Control wells untreated with the extract, (**B**) Test wells treated with the extract.

The OD levels in untreated *Candida* isolates (control) ranged from 0.380 to 1.380, with a mean of 0.690 ± 0.340, while the OD levels in *Candida* isolates treated with *C. longa* extract ranged from 0.350 to 0.490, with a mean of 0.390 ± 0.04, with biofilm inhibition activity ranged from 1.75% to 73.50%, with a mean of 31.77% ±27.06, with significant inhibition *P-value* 0.0015, (Fig. 5, and Table 5).

**Table 5:** Biofilm inhibition activity of *C. longa* extract.

	Biofilm formation positive					
	Mean	SD	Median	Minimum	Maximum	*P-value*
Biofilm formation OD	0.69	0.34	0.53	0.38	1.38	0.0015
Biofilm inhibition activity %	31.77	27.06	25.50	1.75	73.50	
Biofilm inhibition activity OD	0.39	0.04	0.39	0.35	0.49	

Statistically significant at P < 0.05.

Table 6 revealed that the antifungal activity of *C. longa* extract by MIC was directly related to its biofilm inhibition activity with significant relation (*P-value* < 0.0001, = 0.0031, = 0.0010) with significant, moderate, and low activity, respectively.

**Table 6:** The relation between MIC levels of *C. longa* extract and its antibiofilm activity.

	*C. longa* MIC					
	Significant activity		Moderate activity		Low activity	
	Mean	SD	Mean	SD	Mean	SD
Biofilm formation OD	1.15	0.24	0.72	0.41	0.60	0.19
Biofilm inhibition activity %	67.17	6.25	31.88	31.76	25.45	23.39
Biofilm inhibition activity OD	0.37	0.02	0.39	0.03	0.42	0.05
*P-value*	< 0.0001		= 0.0031		= 0.0010	

Statistically significant at P < 0.05.

## Discussion

*Candida* has been associated with a wide range of human infections, morbidity, and mortality. The spectrum of infections ranges from the superficial skin and its appendages to deep-seated or disseminated candidiasis^39^. Recently, the shift from *C. albicans* dominance to non-*albicans*, together with the worldwide reports of increased resistance to antifungals, especially with NAC, is alarming^40,41^.

The FT-IR spectral analysis revealed the presence of various functional groups, confirming the rich phytochemical composition of *C. longa*. The strong O–H and C = O stretching bands signify the presence of curcumin and related polyphenolic compounds known for their antioxidant and anti-inflammatory properties. Similar spectral profiles were reported by Rohman et al. (2015)^42^ and Kusumadewi et al. (2023)^43^, where FT-IR peaks between 3400 and 3200 cm⁻¹ were attributed to hydroxyl groups and those around 1650 cm⁻¹ to carbonyl vibrations of curcuminoids. The observed C–O and C–H stretching bands in the fingerprint region further indicate the presence of terpenoids and essential oil constituents typical of *C. longa* rhizomes^44^. These findings corroborate previous studies^45–47^ that associated such vibrational modes with biologically active secondary metabolites responsible for the therapeutic antifungal properties of turmeric against *Candida sp.*

The findings of the present study underscore a significant epidemiological shift in *Candida* infections, where NAC species surpassed *Candida albicans* in prevalence (67.5% vs. 32.5%). This aligns with global epidemiological data indicating a rising dominance of NAC species in clinical settings, especially *C. glabrata*, *C. krusei*, and *C. tropicalis*^48,49^. Several Egyptian studies similarly reported high NAC prevalence, with Aziz et al. (2024)^50^ reported a notably high prevalence of NAC species, accounting for 63.6% of isolates in adults and 52.7% in pediatric patients, indicating a significant shift toward NAC dominance in both age groups These findings emphasize that the clinical landscape of candidiasis is evolving, likely driven by selective antifungal pressure and shifts in patient populations.

Our data demonstrated that *C. glabrata* was the most frequently isolated species (50%), followed by *C. albicans* (32.5%), *C. tropicalis* (7.5%), *C. krusei* (7.5%), and *C. parapsilosis* (2.5%). This distribution deviates from classical epidemiology, where *C. albicans* predominates, suggesting that hospital settings in Egypt may increasingly mirror global patterns of NAC dominance^51^. The predominance of *C. glabrata* is particularly concerning as it exhibits intrinsic resistance to azoles, complicating therapeutic options^2^. Moreover, the emergence of *C. krusei* intrinsically resistant to fluconazole highlights the impact of widespread azole use on local epidemiology^52^.

Geographic variability in *Candida* epidemiology has been well documented. For example, studies from Asia and Africa report a higher prevalence of *C. tropicalis*^53^, whereas in North America and Europe, *C. glabrata* has emerged as a leading cause of candidemia^1^. Such differences underscore the importance of continuous surveillance and local epidemiological studies to inform treatment policies.

The GTT demonstrated 100% sensitivity and 81.48% specificity, confirming its utility as a rapid screening tool for *C. albicans*. However, its limitations are evident in the potential for false positives with species like *C. tropicalis* and *C. krusei*, necessitating confirmatory methods such as chromogenic media or automated systems like Vitek 2^54^.

Chromogenic agar is an economical and simple method; all the isolates that didn’t show filamentous growth and yielded different colors other than *C. albicans* (green color) were considered NAC spp. For *C. glabrata*, 18(90%) isolates produced the expected white creamy color, while 2 (10%) isolates produced a pale purple color. All 3 *C. tropicalis* isolates (100%), 3 *C. krusei isolates* (100%), and one *C. parapsilosis* (100%) isolate showed the expected reference color (Blue for *C. tropicalis*, purple for *C. krusei*, and white for *C. parapsilosis*). Thus, HiCrome differential agar medium showed an overall accuracy of 92.6% in the case of NAC spp.

In agreement with our results, the sensitivity of chromogenic media in the identification of *Candida spp.* was 96.3% and 97.5% in previously reported studies conducted in Egypt^55^. However, Chromogenic agar could not differentiate *C. albicans* from *C. dubliniensis*, and some spp. have no distinct color on it.

The antifungal susceptibility testing revealed high sensitivity of *Candida* isolates to fluconazole (94.1%). While fluconazole remains widely prescribed due to its oral availability and safety profile, rising resistance globally is limiting its effectiveness^41^. Reports of fluconazole resistance in *C. glabrata* and *C. tropicalis* are particularly alarming, as recurrent and prophylactic azole use exerts selective pressure^11^.

Interestingly, amphotericin B resistance was observed in 20% of isolates (other than *C*. *glabrata*) in our study, primarily among *C. tropicalis* and *C. krusei*. While amphotericin B resistance is generally rare, increasing reports of *C. auris* resistance in clinical settings highlight a growing concern^17^. Amphotericin B remains a cornerstone therapy for invasive candidiasis, but its nephrotoxicity and emerging resistance necessitate safer alternatives or adjunctive therapies^5^.

Comparisons with regional data show some variability. Abouzeid et al., (2023)^56^ reported amphotericin B susceptibility rates exceeding 95% in Egyptian hospitals, while Khairat et al., (2019)^57^ noted azole resistance in 44% of NAC isolates. Such variability reflects differences in hospital settings, antifungal prescribing patterns, and patient populations, reinforcing the need for routine susceptibility testing in clinical practice.

The current study demonstrates that *C. longa* extract exhibited moderate antifungal activity against *Candida* isolates, with an average inhibition zone of 12.33 ± 7.17 mm, although this was significantly smaller than that for fluconazole (28.06 ± 9.01 mm) and non-significantly smaller than that for amphotericin B (14.20 ± 6.76 mm, *P-value* = 0.4942). *C. longa* displayed activity against both *C. albicans* and NAC isolates, indicating broad-spectrum potential.

MIC analysis revealed values ranging from 6.40 to 815 µg/mL, with higher susceptibility observed among *C. albicans* isolates (mean MIC = 183.74 µg/mL) compared to NAC spp. (mean MIC = 280.51 µg/mL). Notably, some isolates demonstrated significant activity with MIC ≤ 10 µg/mL, highlighting *C. longa*’s potential as a potent natural anti-fungal agent. The inverse correlation between inhibition zone diameter and MIC further validated the reproducibility of these results^58^.

Our findings corroborate previous studies reporting *C. longa*’s antifungal effects. Maxwell et al. (2021)^31^ demonstrated potent anti-Candidal activity of curcumin, while more recent work by Tsopmene et al. (2024)^25^ confirmed curcumin’s ability to enhance azole efficacy and inhibit biofilm formation. These findings position *C. longa* as a *Candida*te for adjunctive antifungal therapy, especially in resource-limited settings where drug resistance is prevalent.

A notable aspect of this study is the demonstrated synergistic activity of *C. longa* extract with fluconazole, significantly increasing inhibition zone diameters (31.94 ± 6.99 mm, *P-value* < 0.0001). This suggests that *C. longa* may potentiate fluconazole’s efficacy against susceptible isolates, overcoming some limitations of azole monotherapy.

Also, the combination of *C. longa* with amphotericin B showed significant enhancement, particularly in isolates otherwise resistant to amphotericin B (17.75 ± 7.89, *P-value* = 0.05). These results echo findings by Maxwell et al. (2021)^31^, who reported synergism between curcumin and fluconazole, and Tsopmene et al. (2024)^25^, who observed enhanced antifungal and antibiofilm effects when curcumin was combined with azoles and echinocandins.

Synergistic interactions are clinically valuable as they may reduce the required dosage of antifungal drugs, thereby minimizing toxicity while maximizing efficacy. Moreover, combination therapy may delay or prevent the emergence of resistance, an urgent need in the current post-antifungal era^51,59^.

One of the most significant findings of this study was the *C. longa* extract. Biofilm formation was detected in 40% of isolates, with higher rates among *C. glabrata* and *C. krusei*. *C. longa* significantly reduced biofilm formation by an average of 31.77%, with stronger inhibition observed in isolates with lower MICs.

*Candida* spp. is a highly adaptable microorganism, being able to develop resistance following prolonged exposure to antifungals. Formation of biofilms, which diminish the accessibility of the antifungal, selection of spontaneous mutations that increase expression or decrease susceptibility of the target, altered chromosome abnormalities, overexpression of multidrug efflux pumps, and the ability to escape host immune defenses are some of the factors that can contribute to antifungal tolerance and resistance^11^.

*Candida* biofilms are notoriously resistant to antifungal drugs due to their extracellular matrix, reduced metabolic activity, and efflux pump overexpression^10^. The ability of *C. longa* to disrupt biofilm formation, therefore, represents a promising therapeutic avenue. Our results align with findings by Guimarães et al. (2021)^9^, who reported the antibiofilm potential of medicinal plants, and Tsopmene et al. (2024)^25^, who demonstrated synergistic antibiofilm effects of curcumin with conventional antifungals. Given the role of biofilms in recurrent and device-associated candidiasis, natural products such as *C. longa* may provide a valuable tool to improve treatment outcomes and reduce relapse rates.

The clinical implications of this study are manifold. First, the predominance of NAC spp. Highlights the need for routine species-level identification in clinical microbiology laboratories. Reliance on empirical treatment assuming *C. albicans* predominance is no longer justified, particularly in high-risk hospital units.

Second, the detection of amphotericin B resistance in a subset of isolates emphasizes the importance of antifungal stewardship. Without proper surveillance and rational use of antifungals, the risk of resistance escalation is substantial, as evidenced by the global spread of *C. auris*^17^.

Third, the demonstration of *C. longa*’s antifungal, anti-biofilm, and synergistic properties provides a foundation for exploring natural product-based adjunct therapies. While clinical application requires further validation, our findings highlight the potential role of plant-derived compounds in mitigating antifungal resistance and enhancing current treatment regimens.

## Conclusion

This study revealed a marked predominance of non-*albicans Candida* species, particularly *C. glabrata*, emphasizing the need for vigilant epidemiological surveillance and species-level identification. Antifungal susceptibility testing highlighted continued fluconazole efficacy but concerning resistance to amphotericin B.

*C. longa* extract exhibited moderate antifungal activity, significant anti-biofilm effects, and synergism with fluconazole and amphotericin B, suggesting its potential as a natural adjunctive therapy in managing *Candida* infections. These findings contribute to the growing body of evidence supporting the integration of plant-derived compounds into antifungal strategies, especially in the context of rising drug resistance. While *C. longa* extract demonstrated promising antifungal and antibiofilm activities, this study was limited to in vitro evaluation. Further in vivo and clinical validation is required to confirm its therapeutic potential.

### Recommendations

Regular epidemiological monitoring of *Candida* species and their antifungal resistance patterns should be integrated into clinical laboratory practices to enable early detection of resistant strains and guide appropriate treatment strategies. Establishing antifungal stewardship programs is equally important to ensure the rational use of azoles and amphotericin B, thereby minimizing unnecessary prescriptions and reducing selective resistance pressure. In addition, the development of adjunctive therapies using natural compounds such as *Curcuma longa* offers promising potential; clinical trials combining *C. longa* formulations with fluconazole may enhance therapeutic efficacy and decrease toxicity. Addressing biofilm formation is another key priority, as biofilm-associated infections, particularly those linked to medical devices, often exhibit high resistance to conventional treatments. The entire biomass of the biofilm, including the extracellular matrix and viable and non-viable cells, was measured using crystal violet staining. Despite not directly measuring metabolic activity, this technique is still a commonly used and standardised assay for research on biofilm inhibition. The effects of Curcuma longa extract on biofilm cell viability may be further clarified by future research using metabolic viability tests like XTT or resazurin.

Finally, molecular and mechanistic research on the antifungal and antibiofilm activities of *C. longa* is needed to better understand its mode of action and support the development of new, more effective antifungal drugs.

## Data Availability

The datasets used and analyzed during the current study are available from the corresponding author on reasonable request. All data supporting the conclusions of this article are included within the article.
